# Tail state mediated conduction in zinc tin oxide thinfilm phototransistors under below bandgap optical excitation

**DOI:** 10.1038/s41598-021-98339-4

**Published:** 2021-09-24

**Authors:** Soumen Dhara, Kham M. Niang, Andrew J. Flewitt, Arokia Nathan, Stephen A. Lynch

**Affiliations:** 1 School of Physics and Astronomy, Cardiff University, Cardiff, CF24 3AA UK; 2 Electrical Engineering Division, Department of Engineering, University of Cambridge, Cambridge, CB3 0FA UK; 3 Darwin College, University of Cambridge, Cambridge, CB3 9EU UK; 4 Present Address: Faculty of Science, Sri Sri University, Cuttack, 754006 India

**Keywords:** Materials for devices, Electronic devices, Optical materials and structures

## Abstract

We report on the appearance of a strong persistent photoconductivity (PPC) and conductor-like behaviour in zinc tin oxide (ZTO) thinfilm phototransistors. The active ZTO channel layer was prepared by remote plasma reactive sputtering and possesses an amorphous structure. Under sub-bandgap excitation of ZTO with UV light, the photocurrent reaches as high as ~ 10^−4^ A (a photo-to-dark current ratio of ~ 10^7^) and remains close to this high value after switching off the light. During this time, the ZTO TFT exhibits strong PPC with long-lasting recovery time, which leads the appearance of the conductor-like behaviour in ZTO semiconductor. In the present case, the conductivity changes over six orders of magnitude, from ~ 10^−7^ to 0.92/Ω/cm. After UV exposure, the ZTO compound can potentially remain in the conducting state for up to a month. The underlying physics of the observed PPC effect is investigated by studying defects (deep states and tail states) by employing a discharge current analysis (DCA) technique. Findings from the DCA study reveal direct evidence for the involvement of sub-bandgap tail states of the ZTO in the strong PPC, while deep states contribute to mild PPC.

## Introduction

Coupling, or interaction between constituent materials, in ternary/quaternary compounds or hybrid composites has the potential to unlock exceptional properties which are superior to those of the pristine binary materials. Furthermore, the bandgap energy can be easily tuned by changing the compositional stoichiometry of the ternary/quaternary oxide semiconductors according to requirements. Recently, amorphous compound oxide semiconductors (AOS) have unlocked new electronic and optoelectronic functionality with the development of transparent and flexible devices^1–6^. The initial breakthrough came through the first demonstration of indium gallium zinc oxide (IGZO) thinfilm transistors (TFTs) by the Hosono group in 2004^7^. IGZO based TFTs have been used in flat panel displays^8^ and flash memory applications^9^ due to its high electron mobility, strong light sensitivity, and ultralow power consumption. However, many research laboratories have recently ramped up research on alternative AOS TFT materials to avoid over-reliance on indium^2,10,11^. Similar to IGZO, a simple ternary oxide, zinc-tin-oxide (ZTO), is also very sensitive to light and highly conductive^12^. In addition, ZTO has better chemical stability against oxidation and chemical etching^13^. Recently, TFTs made with ZTO active channels have been demonstrated, and show promising electrical characteristics^6,14–16^. The reported results/performance of ZTO based TFTs indicate that it could be a good competitor to IGZO based TFTs. However, the photo-electrical properties of ZTO TFTs have been less well-explored, and there are very few reports in the literature^17–19^. Oxide semiconductors suffer from one critical issue, namely persistent photoconductivity (PPC), which hinders successful commercial applications in photosensing. In order to resolve this issue, various materials and approaches have been introduced and studied over the past few years^18,20–22^. However, a straightforward and effective technology applicable to all AOS has yet to develop. Although, it is believed that oxygen vacancies are responsible for the PPC effect, direct evidence for this has yet to emerge. Consequently, the lack of in-depth understanding of PPC in binary and ternary oxides has impeded its useful exploitation in optoelectronics. A detailed study on the photoexcited transport and sensing properties of the ZTO would be helpful to the research community for the development of next-generation flexible optoelectronic devices.

In this work, we investigated photoexcited electrical transport characteristics of ZTO TFTs with different concentrations of tin for a range of different photon energies. We observed strong PPC with a high value of the photo-to-dark current ratio (sensitivity) and appearance of the conductor-like behaviour in the ZTO semiconductor. Under the illumination of UV light at 365 nm, with photon energy just below the band-to-band excitation energy of ZTO, the sensitivity reaches as high as ~ 10^7^. With the exposure of UV light for 15 min, we observed a transition where semiconductor ZTO shows conductor-like behaviour, with high photosensitivity and strong PPC (lasting up to a month). To investigate further the observed strong PPC, we studied the material’s photo-response, by illuminating TFTs at different photon energies in the sub-bandgap region. We extracted the ZTO defect density, and density of sub-bandgap states (DOS), by employing a discharge current analysis (DCA) technique. The DCA results show that ZTO TFTs with higher tin concentration exhibit higher sub-bandgap tail states. A correlation between photoexcitation energy and DOS distribution is discussed in detail, leading to a direct evidence of the involvement of tail states of the ZTO in the observed strong PPC.

Our report is structured as follows: in the first section we discuss the relevant material properties and the corresponding device output characteristics measured on devices fabricated from this material. The material roughness was measured by AFM. The representative IV characteristics allowed us to extract, the field effect mobility (μ_FE_), and the sub-threshold slope (SS). The material bandgap (E_g_) was established from a Tauc plot, whereby the absorption coefficient was extracted from the transmittance spectrum. In the next section, we consider the photoexcited transfer characteristics. The IV characteristics were recorded both under above bandgap UV illumination, and under below bandgap visible illumination. Knowledge about the distribution of the sub bandgap defect states was predominantly established from the below bandgap illumination experiments. Next we describe the transient response characteristics by considering the evolution of the drain-source current (I_DS_) over time under fixed gate-source and drain source voltage (V_GS_ and V_DS_ respectively). In the next section, we attempt to correlate the observed device behaviour with what we know about the material properties. Here XPS was used to establish the oxygen content of the material. The relative concentrations of chemically bonded O^2−^ ions and oxygen vacancies (Ov) was obtained by deconvolving the main oxygen peak in the X-ray spectrum. Finally, we describe how the discharge current analysis (DCA) technique was employed to establish the density and distribution of sub-bandgap defect states near the conduction band edge. We bring our report to a close with a discussion describing our interpretation of the findings, and a concluding section. Some of the experimental techniques used for this work are further expanded upon in the Methods section.

## Results and discussion

### Representative material and device output characteristics

**Figure 1:**
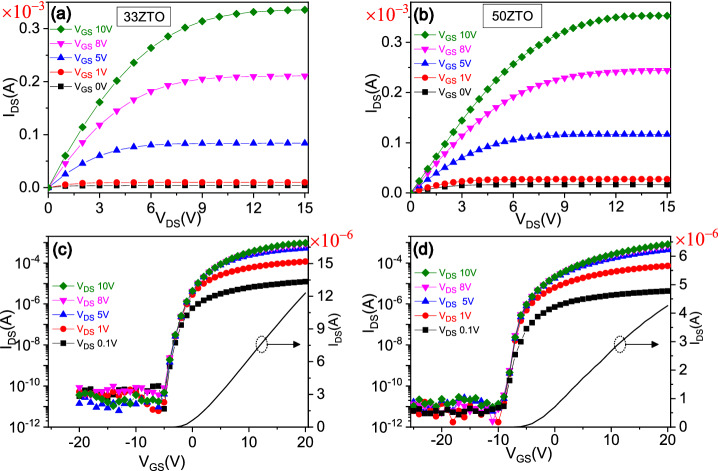
The output characteristics (I_DS_ vs. V_DS_) of ZTO TFTs for the sample (**a**) 33ZTO and (**b**) 50ZTO measured at different V_GS_. In (**c**) and (**d**), the transfer characteristics (I_DS_ vs. V_GS_) of ZTO thinfilm transistors for the sample 33ZTO and 50ZTO, respectively. In both the TFTs, active semiconductor channel W/L ratio was fixed to 38.

The active semiconductor channel of both sets of fabricated TFTs (33ZTO, contains 33 at% of tin and 50ZTO, contains 50 at% of tin) possess amorphous crystal structure, as expected. The root mean square (RMS) surface roughness of the as prepared thinfilm is estimated from AFM images (data not shown). A small surface roughness of 1.5 nm indicates a smooth surface of the thinfilm. Details of microstructural and morphological characterization results can be found in the previous report^23^. The electrical measurement data of ZTO TFT shows the typical output characteristics (I_DS_ vs. V_DS_) and transfer characteristics (I_DS_ vs.V_GS_) of a field effect transistor, with distinct linear and saturation regions (Fig. 1). Several important characteristic parameters of the ZTO TFTs are extracted from the gate transfer graph for both the 33ZTO and 50ZTO TFTs. Based on standard field effect transistor theory^24^, the field effect mobility (μ_FE_) and sub-threshold slope (SS) are extracted from a linear fit to the experimental I_DS_ vs. V_GS_ data at V_DS_ = 0.1 V in the linear region and log-scale plot, respectively using following equations,

1 $$\upmu_{{{\text{FE}}}} = \frac{{\frac{{\partial {\text{I}}_{{{\text{DS}}}} }}{{\partial {\text{V}}_{{{\text{GS}}}} }}}}{{{\text{C}}_{{{\text{ox}}}} \left( {{\text{W}}/{\text{L}}} \right){\text{V}}_{{{\text{DS}}}} }}$$

2 $$\mathrm{SS}=\frac{{\mathrm{dV}}_{\mathrm{GS}}}{\mathrm{dlog}({\mathrm{I}}_{\mathrm{DS}})}$$

where C_ox_, W, L, I_DS_, V_DS_ and V_GS_ are gate oxide capacitance (~ 18 nF/cm^2^), channel width (1900 μm), channel length (50 μm), drain-to-source current, drain-to-source voltage and gate-to-source voltage, respectively. The estimated μ_FE_ and SS are found to be 9.07 cm^2^/V s and 0.92 V/dec, with a threshold voltage (V_th_) of − 0.1 V for the 33ZTO TFT. In the 50ZTO TFT, which contains a higher tin concentration, it possesses lower μ_FE_ of 6.07 cm^2^/V s and SS of 0.65 V/dec with a V_th_ of − 3.9 V. Here we note that the magnitude of the V_th_ is estimated from the gate transfer data measured at a voltage sweep rate of 0.33 V/s. In oxide-based semiconductor TFTs, measurement of V_th_ may be affected by voltage sweep rate due to the charge trapping effect^25,26^. The lower value of mobility in 50ZTO indicates that the density of charge trapping centers is higher in 50ZTO than the 33ZTO TFT. The estimated magnitudes of each of these parameters are comparable or slightly better than the rival IGZO based TFTs and ZnO or SnO_2_ based TFTs^7,27–29^. So, both the TFTs can operate in the ON state at zero gate bias. However, the device requires a negative gate voltage to switch off the TFTs which increases the power consumption. Ideally, to reduce power consumption, it would be necessary to achieve a positive threshold voltage close to zero voltage.

**Figure 2:**
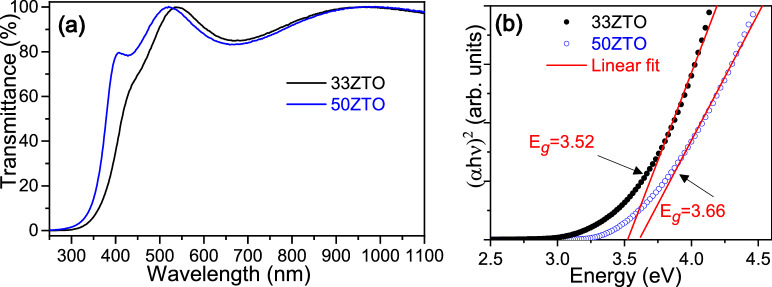
(**a**) UV–Vis transmission spectra and (**b**) calculation of the bandgap energy of the ZTO thinfilms on Corning glass substrate with an elemental composition identical to semiconductor channels of 33ZTO and 50ZTO TFTs.

To study the photoconductivity of semiconducting material, the first requirement is to know the exact bandgap energy of that material. The optical bandgap energy (E_g_) of the ZTO thinfilm is estimated from its UV–visible transmission spectrum, as shown in Fig. 2a. Transmittance data of the ZTO thinfilms shows a highly transparent film with transparency above 85% in the visible-to-NIR region. The bandgap energy is calculated from the standard Tauc plot relation assuming a direct allowed transition. A linear fit to the (αhν)^2^ vs. energy (hν) and calculated E_g_ are shown in Fig. 2b. The estimated bandgap energies are found to be 3.52 eV and 3.66 eV for the ZTO thinfilms with 33 at% and 50 at% of tin concentrations, respectively. This provides evidence that ZTO thinfilms with a higher concentration of tin show larger bandgap than the case of lower tin concentrations, according to the Burstein–Moss effect. The electrical characterization data of the TFTs and Hall effect measurement of the ZTO thinfilms (data not shown, see Ref.^23^) show higher carrier concentration for higher Sn content sample thus supporting the bandgap widening hypothesis according to the Burstein–Moss effect. The estimated order of magnitude of carrier concentration of the ZTO thinfilms from Hall Measurement is 10^17^/cm^3^. Furthermore, E_g_ appears in a range between the bandgap energies of crystalline ZnO (~ 3.37 eV)^30^ and SnO_2_ (~ 4.05 eV)^31^ films, as expected.

### Photoexcited transfer characteristics

**Figure 3:**
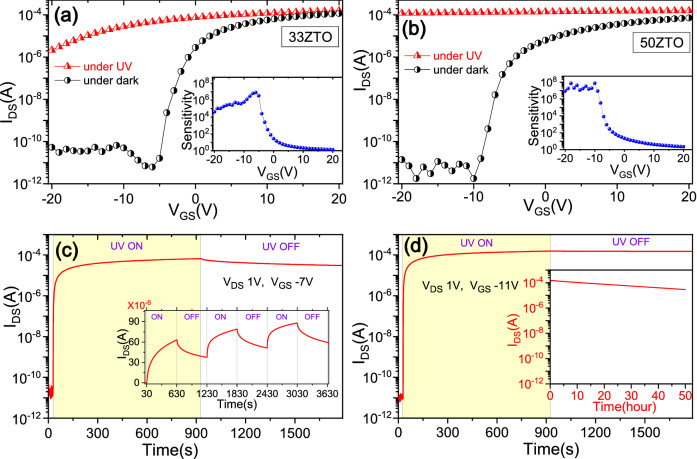
The transfer characteristics of (**a**) 33ZTO and (**b**) 50ZTO TFTs at V_DS_ = 1 V measured in dark and under UV light (at 365 nm) exposure. The sample was exposed to UV light for 5 min at an intensity of 40 mW/cm^2^ before starting the photocurrent–voltage measurement. Insets show the sensitivity (photo-to-dark current ratio) as a function of V_GS_. In (**c**) and (**d**), the transient response of the drain-to-source current for ZTO photo-TFTs under a single UV light pulse of 15 min for the sample 33ZTO and 50ZTO, respectively. The inset in (**c**) shows photoresponse of 33ZTO under a periodic UV pulse (10 min). The inset in (**d**) shows extended measurement (up to 50 h) of drain-to-source photocurrent after switching off the UV light.

To study the photoexcited transfer characteristic of ZTO TFTs, the device was exposed to UV light at 365 nm (intensity ~ 40 mW/cm^2^) for 5 min before measuring I_DS_ vs. V_GS_ data. In order to concentrate on the defect-induced photoconductivity study of the ZTO TFTs, the photon energy was limited to below the bandgap energy of ZTO (3.52–3.66 eV). Under illumination, the photogenerated drain-to-source current of ZTO TFTs increases from a few picoamps to miliamps in the accumulation region, as shown in Fig. 3a,b and operates as a thinfilm phototransistors. In case of the 33ZTO TFT, at a gate bias of − 6 V, the drain-to-source current increases from 6.2 × 10^−12^ to 4.7 × 10^−5^ A, leading to a sensitivity (defined as the photo-to-dark current ratio at the same drain and gate voltages) up to 10^6^. Similarly, in the 50ZTO TFT at a gate bias of − 10 V, the drain-to-source current increases from 1.7 × 10^−12^ to 1.3 × 10^−4^ A, leading to a sensitivity of 10^7^. The sensitivity vs. gate voltage plot shows a strong gate-tuning effect, where the sensitivity gradually increases with decreasing applied gate bias, and saturates at a gate bias below the flatband voltage (V_FB_). Furthermore, upon light illumination, a large negative shift in the threshold voltage (ΔV_th_ > 16 V) is observed from both the TFTs and the devices remain in the ON state up to − 20 V (measurement limit) of gate bias. The negative shift in the threshold voltage was observed previously in most of the AOS, where the magnitude of the negative shift depends on the light intensity and light stress time^20,32–34^. Both the TFTs exhibit excellent photoconductivity, with a very high value of sensitivity, which is one of the important criteria for development of the thinfilm transistor-based smart UV sensors.

### Transient response characteristics

Next, we studied the time response of the photo-TFTs by measuring transient drain-to-source current in the accumulation region in light ON and OFF conditions, as shown in Fig. 3c,d. Upon UV illumination (at 365 nm), the photocurrent increases exponentially with time. However, in the present case for both the TFTs, the photocurrent neither saturates to a high current value nor does it reset to the initial dark current reading after 15 min of light exposure followed by no further illumination. After 15 min of decay measurements (after switching off the light), the photocurrent decreases to 54.4% of the maximum value for the 33ZTO TFT and only 2.9% for the 50ZTO TFT (which is an exceptionally slow recovery process). Our observations confirm that the ZTO TFTs exhibit a strong PPC effect with long-lasting recovery time. Transient photocurrent measurement under a periodic UV illumination pulse (10 min), as shown in the inset of Fig. 3c further confirms the repeatability of the observed long-lasting PPC in the ZTO TFTs. The maximum photocurrent in the next cycle is increased, compared to the previous cycle, because of the incomplete increase and recovery of the photocurrent during the measurement cycle. To estimate the reset time (defined as the time required for recovery to 37% (1/e) of the maximum photocurrent), decay measurements were performed for longer time periods. Extended measurement of drain-to-source photocurrent (up to 50 h) for the 50ZTO TFT is shown as an inset in Fig. 3d. The reset time is estimated to be 55.8 min and 29.7 h for the 33ZTO and 50ZTO TFTs, respectively. Here we note that 50ZTO TFT exhibits a strong PPC with recovery time ~ 30 times larger than the 33ZTO TFT. The mathematical extrapolation of the photocurrent decay data for the 50ZTO TFT indicates that this persistent photocurrent can be potentially maintained for up to a month. This long-lasting PPC far exceeds all previously reported results from other AOS^18,20,21^. We conclude from this, that tin concentration in the ZTO ternary oxide modulates the photosensitivity as well as PPC recovery time. The observed long-lasting PPC at a sub-bandgap photoexcitation implies that most of the excess electrons are generated from optically irreversible states. These irreversible states could be sub-bandgap deep defects states or tail states. In AOS, oxygen vacancy related defects (V_o_) are very common. These defects become ionised under optical illumination (V_o_ → V_o_^+^/V_o_^2+^ + e^−^/2e^−^) providing free electrons^20,35,36^. It is believed that PPC is associated with these ionised oxygen defects, which oppose the recovery process by charge localisation inducing a slowdown in the reaction, V_o_^2+^ + 2e^−^ → V_o_. However, in the present case, there is a vast difference in the long-lasting PPC between 33ZTO TFT and 50ZTO TFT, which indicates that in addition to oxygen vacancy defects, another irreversible state is also responsible. This result agrees with the XPS study (discussed later) which reveals that oxygen vacancy is higher in 33ZTO than the 50ZTO.

The strong PPC with a high value of sensitivity (~ 10^7^) in the 50ZTO TFT causes the semiconducting channel to become a conductor. In the present case, the conductivity of the 50ZTO compound in the dark is estimated as ~ 10^−7^/Ω/cm. Upon UV exposure, conductivity increases to 0.92 Ω/cm, which is comparable to the conductivity of conductors like semi-metals, and remains at a high value for a long time after switching off the UV light After 50 h the conductivity was measured to be 0.16/Ω/cm. The UV exposure thus causes the 50ZTO sample to undergo a transition (semiconductor ZTO to conductor-like ZTO). We believe the highly photo-responsive characteristic of the ZTO semiconductor, combined with the semiconductor-to-conductor transition, may facilitate the favourable exploitation of PPC in the next generation smart optoelectronic devices.

**Figure 4:**
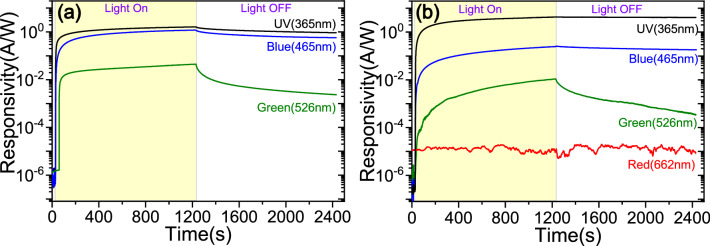
Transient response of responsivity (photocurrent generated per unit of applied light intensity) of the ZTO TFTs for the sample (**a**) 33ZTO and (**b**) 50ZTO measured at different photoexcitation energy: UV (at 365 nm); blue (at 465 nm); green (at 526 nm); red (at 662 nm).

To investigate further the observed strong PPC effect, we studied photoresponse of the TFTs in light ON and OFF conditions, for different photon excitation energies in the sub-bandgap region. For a comparative study, the measured photocurrent is converted to the responsivity by normalising against the light intensity of the specific light source. Figure 4 and Table 1 show the transient responsivity for the 33ZTO and 50ZTO TFTs measured under the illumination of UV (at 365 nm), blue (at 465 nm), green (at 526 nm) and red (at 662) lights. The transient photocurrent data under the illumination of blue and green light show similar characteristics to the responsivity measured under UV illumination. Under illumination by red light with photon energy far below the bandgap of 50ZTO, no notable photo-generation of charge carriers appears (Fig. 4b) due to an absence of sufficient activation of defect states. Similar behaviour for the 33ZTO under illumination by red light is expected. More interestingly, the PPC effect improves when the active channel is illuminated by blue/green light, which means there is a comparatively faster photocurrent decay during recovery. Under excitation by blue light, the reset times are 21.2 min and 70.1 min for the 33ZTO and 50ZTO TFTs respectively, which is far better than the case of photoexcitation with UV light. The reset time is further improved under excitation by green light (53 s for 33ZTO and 90 s for 50ZTO). The maximum responsivity for the 33ZTO after 20 min of light exposure gradually decreases with a decrease in photon energy, as expected (1.592 A/W under UV, 1.197 A/W under blue and 0.045 A/W under green). In contrast, the 50ZTO TFT responsivity is 4.350 A/W at UV and then drastically reduces with decreasing photon energy (0.259 A/W at blue and 0.011 A/W at green). It is well accepted that V_o_ defects are responsible for the slow decay process in the oxide semiconductors and decay times are comparable at different excitation energies. Our experimental results are also in agreement with this mechanism. Much faster comparative reset time can be expected under illumination with light of photon energy above the bandgap energy of the ZTO. Faster reset times of the order of few sec to several sec have been reported by different research groups from the high quality ZTO thinfilms at wavelength < 350 nm (photon energy is higher than the bandgap energy of the ZTO)^19,37^. Relatively low sensitivity and slower photoresponse were observed when illuminated with light at wavelength 450 nm.

**Table 1:** Extracted parameters from the photoresponse characteristics (Fig. 4) of the ZTO thinfilms.

Sample	Excitation wavelength (nm)	Sensitivity	Responsivity (A/W)	Reset time (min)
33ZTO	365	10^6^	1.592	55.8
	465	–	1.197	21.2
	526	–	0.045	0.9
50ZTO	365	10^7^	4.350	1782.2
	465	–	0.259	70.1
	526	–	0.011	1.5

It is observed that the photoresponse of the amorphous oxide semiconductor TFTs operated in negative bias under illumination stress (NBIS) depends on the photon energy and the intensity (mW/cm^2^) of the light source. In a report by Chowdhury et al.^38^, the well-performing and stable IGZO TFTs under NBIS with a 365 nm wavelength and only 0.7 mW/cm^2^ of light intensity, exhibited a V_th_ shift as high as ~ − 10 V is reported. Fernandes et al.^39^ reported a V_th_ shift of − 3.0 V under NBIS with a wavelength of 420 nm from a ZTO TFT annealed at 300 °C. It is likely that the light intensity is relatively small (the actual value is not mentioned) in this case but enough to cause a V_th_ shift after 1 h of exposure. In the present case, the photoresponse measurement under UV was carried out at a light intensity of 40 mW/cm^2^. In addition, ZTO thinfilms were grown at room temperature with low sputtering power. After a high temperature annealing at 500 °C the resulting trap density is of the order of 10^12^/cm^2^/eV which is still higher than the trap density present in the samples prepared by Fernendes et al. This suggests that the 500 °C annealed devices do not necessarily result in better/more stable devices.

### Influence of material properties on photocurrent behaviour

**Figure 5:**
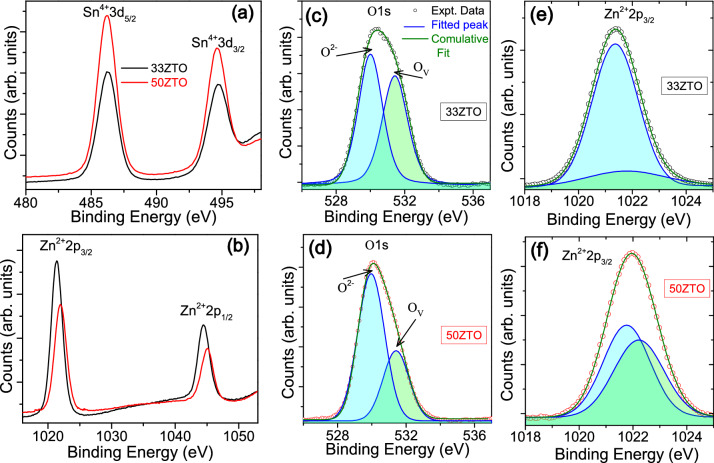
XPS binding energy spectra of the ZTO thinfilms with an elemental composition identical to semiconductor channel of 33ZTO and 50ZTO TFTs for the core level of: (**a**) Sn3d, (**b**) Zn2p, (**c** and **d**) O1s and (**e** and **f**) Zn^2+^2p_3/2_. Individual component (lattice oxygen and oxygen vacancy) in the broad and asymmetric O1s spectrum and individual component (lattice Zn in the oxide form and Zn interstitials) in the Zn^2+^2p_3/2_ spectrum are identified after fitting with multiple Gaussian peaks. Solid green line represents the combined fitting of the sum of all the individual components (blue line).

To analyse the relationship between the observed photocurrent features and oxygen vacancy content, we performed an in-depth investigation on the XPS spectra for the O1s core level. Figure 5 shows the X-ray photoelectron spectroscopy (XPS) binding energy spectra for the core levels of Sn3d, Zn2p and O1s measured from 33 and 50ZTO thinfilms. The XPS data for the Sn3d core level exhibits a peak doublet at 486.3 and 494.8 eV corresponding to Sn^4+^3d_5/2_ and Sn^4+^3d_3/2_, respectively^40^. Similarly, for the Zn2p core level, a doublet with peaks centered at 1021.4 and 1044.4 eV is observed, corresponding to Zn^2+^2p_3/2_ and Zn^2+^2p_1/2_, respectively. We noted that the integrated peak area of the Sn3d doublet increased from 33 to 50ZTO thinfilms, whereas it is reduced for the Zn2p doublet, as expected due to an increase in tin concentration. The broad and asymmetric peak of the O1s core level was deconvoluted, giving two individual components with peaks centered at 530.0 and 531.4 eV. The lower binding energy peak, O^2−^, represents oxygen ions connected with metal ions, and the higher binding energy peak, O_V_, is associated with oxygen ions that are in oxygen deficient regions within the matrix of ZTO (oxygen vacancy, V_O_)^41^. Details of information extracted from the XPS data and the respective identity of the peak are presented in Table 2. The trace of the absorbed water moisture (H_2_O, appears at ~ 532.7 eV) was checked during deconvolution and no such peak observed. As the ZTO thinfilms underwent post-growth annealing at a higher temperature (at 500 °C), there is also a remote possibility of the presence of water moisture on the surface of the thinfilms. When the spectra of the O1s core level for 33ZTO and 50ZTO are compared, the 33ZTO thinfilm shows higher intensity for oxygen vacancies. The relative oxygen vacancy content is estimated by taking ratio of integrated peak area of peak 2 (O_V_) to total area of peak 1 (O^2−^) and peak 2, and it changes from 39.6 to 32.5% with the increase of tin concentration from 33 to 50 at%. The XPS data reveals that 33ZTO contains more V_O_ states than the 50ZTO thinfilm. The oxygen vacancies in the amorphous oxides are believed to be responsible for generating carriers in the depletion region and have a strong influence on the conductivity^28,42^. Similarly, Zn^2+^2p_3/2_ XPS peak was deconvoluted and best fit achieved with two individual components with peaks centred at 1021.4 and 1021.8 eV for 33ZTO thinfilm, and 1021.8 and 1022.2 eV for 50ZTO thinfilm. The lower energy and higher energy components represent lattice Zn in its oxide form and Zn interstitials (Zn_i_), respectively^43,44^. The relative content of Zn_i_ defect is estimated from the integrated peak area of peak 1 and peak 2 of Zn^2+^2p_3/2_ XPS peak, and it increases from 10.5 to 47.2%. The analysis of the XPS data indicates a sharp increase of Zn_i_ defects from 33 to 50ZTO thinfilms. In other words, we can say that the 50ZTO thinfilm content four times higher concentration of Zn_i_ defects than the 33ZTO thinfilm. Here we note that deconvolution of spectra of Zn2p and O1s core levels shows no traces of hydroxyl or hydrogen on the surface of the sample. The formation energy of neutral Zn_*i*_ is 6.95 eV^45^, which is the second-lowest energy amongst all the other commonly found native defects in ZnO. This supports the hypothesis for a high probability of Zn_i_ defects formation. In common with the V_O_ defects (deep donors), Zn_i_ also acts as trap centers (shallow donors) under voltage bias. We note however, that if oxygen vacancies are only responsible for the observed photoresponse features in ZTO TFTs (here, photon energy is not sufficient to create band-to-band electron–hole pairs), higher photocurrents and long-lasting PPC would be expected in 33ZTO rather than 50ZTO, which is the opposite of what we observe. Therefore, considering only the involvement of oxygen vacancy defects, which are usually distributed in the sub-bandgap region of 0.5–1.3 eV below the conduction band (CB), fails to explain the observed photoresponse and PPC in the ZTO TFTs. Contribution from Zn_i_ defects to the PPC of the ZTO cannot be ignored.

**Table 2:** Details of the fitting parameters for Sn3d, O1s and Zn2p core level XPS spectra of zinc tin oxide thinfilms. A_1_ and A_2_ represent integrated peak areas of the peak 1 and peak 2, respectively.

Sample	Sn3d_5/2_ (eV)	O1s			Zn2p_3/2_		
		Peak 1 (eV)	Peak 2 (eV)	$\frac{{\mathrm{A}}_{2}}{{\mathrm{A}}_{1}+{\mathrm{A}}_{2}}$	Peak 1 (eV)	Peak 2 (eV)	$\frac{{\mathrm{A}}_{2}}{{\mathrm{A}}_{1}+{\mathrm{A}}_{2}}$
33ZTO		530.0	531.42	0.396	1021.37	1021.77	0.105
50ZTO	486.20	529.96	531.40	0.325	1021.78	1022.22	0.472
Identity	*Sn–O*	*O*^*2−*^	*V*_*O*_	*–*	*Zn–O*	*Zn*_*i*_	–

### Density and distribution of defects states through discharge current analysis

**Figure 6:**
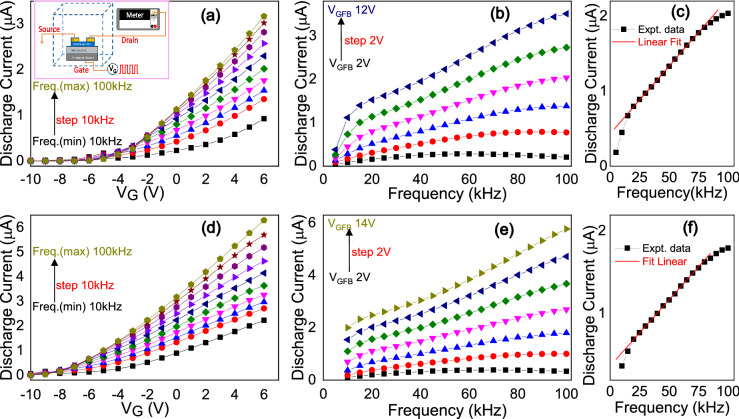
Measured discharge current data, as a function of pulse gate voltage pulse (V_G_) for different pulse frequency (**a**) and as a function of pulse gate voltage frequency for different V_GFB_ (= V_G_ − V_FB_) (**b**), for the sample 33ZTO TFT. The inset shows a schematic diagram of the overview of the discharge current analysis technique with electrical connections. In (**c**), the representative linear fitting to the discharge current-gate pulse voltage frequency experimental data. In (**d**–**f**) measured discharge current data of the same for another TFT, 50ZTO.

In order to investigate further on the underlying physics of the observed photoresponse, we exploited the DCA technique^46,47^ which is a modified charge pumping method (CPM)^48,49^. DCA is a powerful and effective method to extract the distribution of the DOS near the CB in oxide semiconductors in fabricated TFTs. A schematic diagram of the DCA measurement setup with circuit connection is shown as an inset in Fig. 6a. In the DCA method, a periodic voltage pulse with 50% duty cycle at a given frequency is fed into the gate and the average discharge current is measured at the drain following a delay of one second. When the gate pulse amplitude is ramped up above V_FB_, along with the population of the intrinsic bulk carriers (here electrons), the defect sites at the interface and in the active ZTO layer charge up. The charging times of the defect sites were found to be between μs and ms depending on the types of defect sites involved. When the gate pulse is ramped down, populated intrinsic carriers quickly discharge through drain and source. However, various trap sites in the sub-bandgap region of the active ZTO layer slowly discharge and release electrons over a time period of ms up to few seconds. The tail portion of the discharge current is mainly associated with charge released from the sub-bandgap defect sites in the ZTO layer together with contributions from other trap sites in the ZTO-dielectric interface. This tail portion of the discharge current strongly depends on the applied pulse frequency, which shows a linear dependence in the low-frequency regime and then becomes saturated at a very high frequency. Using this method, a signature of the trap sites in the ZTO layer can be extracted from the slope of the discharge current-frequency data.

Figure 6 shows the measured discharge current from the 33ZTO and 50ZTO TFTs as a function of gate voltage and pulse frequency according to the DCA method, as discussed earlier. One can see that discharge current gradually increases as a function of gate voltage (Fig. 6a,d), and also as a function of frequency (Fig. 6b,e), as expected. Furthermore, a notable discharge current is observed only when V_GFB_ (= V_G_ − V_FB_) is positive. As the discharge current saturates at a pulse frequency of 120 kHz, the discharge current vs. frequency measurement was limited to 100 kHz. The trap/defects sites density is extracted from the slope, $\left( {{{\partial I} \mathord{\left/ {\vphantom {{\partial I} {\partial f}}} \right. \kern-\nulldelimiterspace} {\partial f}}} \right)$, in the linear region using the following equation^46,47^

3 $$N_{defect\;site} \left( {\# /{\text{cm}}^{3} } \right) = \frac{2}{k} \cdot \frac{{{{\partial I} \mathord{\left/ {\vphantom {{\partial I} {\partial f}}} \right. \kern-\nulldelimiterspace} {\partial f}}}}{V \cdot q}$$

where N_defect site_ represents the density of defect sites in the sub-bandgap region in the ZTO semiconductor, *k* is the charge loss factor, V is the active volume of the semiconducting layer and q is the elemental charge. The slope is estimated from the linear fitting to the discharge current vs. frequency data for different V_GFB_ at a step of 1 V, and a representative linear fitting for the 33ZTO and 50ZTO TFTs are shown in Fig. 6c,f.

**Figure 7:**
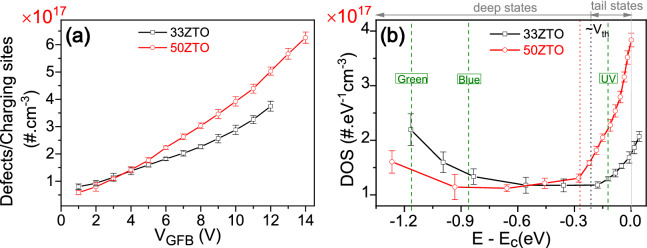
(**a**) The extracted sub-bandgap defects/charging sites density as a function of V_GFB_ (= V_G_ − V_FB_) for 33ZTO and 50ZTO TFTs. (**b**) The extracted density of sub-bandgap states (DOS) versus energy with reference to the conduction band minima. The green dashed lines represent photoexcitation energy levels for the 33ZTO at UV, blue and green light excitations.

The extracted density of the sub-bandgap defect/charging sites for the oxide TFTs, as shown in Fig. 7a gradually increases when increasing the gate pulse amplitude. A defect density on the order of ~ 10^17^/cm^3^ is obtained from both the TFTs, which is comparable to other amorphous oxide semiconductors^35,50,51^. The energy distribution of the sub-bandgap defects (DOS) in the sub-bandgap region is calculated by converting the gate amplitude to the surface potential energy. The surface potential, φ_s_ as a function of V_G_ is calculated by integrating the following equation^52^,

4 $${\varphi }_{s}={\int }_{{V}_{FB}}^{{V}_{Gs}}\left(1-\frac{{C}_{g}({V}_{GS}^{^{\prime}})}{{C}_{ox}}\right)d{V}_{GS}^{^{\prime}}$$

Figure 7b shows the extracted density profile of the sub-bandgap defect sites, which is exponentially distributed in energy, as expected. Interestingly, a significantly high density of tail states extending up to ~ 0.3 eV below the CB is observed in 50ZTO TFT. However, the oxygen vacancy related deep states defect density is higher in 33ZTO TFT than the 50ZTO TFT which is consistent with the XPS analysis results. We have previously reported the direct measurement of the density of sub-bandgap states by photothermal deflection spectroscopy (PDS) and photoconductive laser spectroscopy, which are very sensitive optical absorption methods^53,54^. These measurements shows that the 33ZTO has a higher density of deep states than the 50ZTO. In contrast, 50ZTO film has a higher density of tail states (higher Urbach energy) than the 33ZTO. Even though the Urbach tail states by PDS method cannot distinguish between the conduction band tail and valence band tail, the density of sub-bandgap states measured by PDS is in agreement with the density of sub-bandgap states extracted by the DCA technique. As the transient photocurrent was measured in accumulation mode of the TFT at a gate voltage V_GS_ < V_FB_, the photo-excited charge carriers are the sole source of observed high drain-to-source current. The photoexcitation energy levels of the UV, blue and green light excitations for the 33ZTO TFT (*E*_*g*_ = 3.52 eV) are marked by green dashed lines in Fig. 7b at − 0.12, − 0.85 and − 1.16 eV, respectively. In the case of 50ZTO TFT (*E*_*g*_ = 3.66 eV), the reference levels are at − 0.26, − 0.99 and − 1.30 eV for the UV, blue and green light excitations, respectively. If we compare the DOS and the generated photocurrent for both sets of TFTs, a higher DOS results in higher photogenerated current at a specific photoexcitation level. In the present case, under excitation by blue light, a 10.0% increment of the DOS (deep states) in 33ZTO TFTs leads to a factor of four enhancement (~ 4.34) in the photocurrent compared to the 50ZTO TFT. Similarly, under excitation by UV light, an increment of 7.4% of the DOS (tail states) in 50ZTO TFT leads to a factor of two enhancement (~ 2.63) in the photocurrent compared to the 33ZTO TFT. The sub-bandgap DOS extracted from the DCA technique thus provides direct evidence of the involvement of tail states in the ZTO when excited with UV light, and deep states when excited with blue/green light. Usually, the tail states are situated in the vicinity of the CB within 0.1 eV range. Involvement of the tail states is thus associated with high photosensitivity and strong PPC with long-lasting recovery time, while the deep states are associated with comparatively lower photosensitivity with mild PPC.

## Discussion

It is known that, upon photoexcitation with sufficient photon energy in oxide semiconductors, that the neutral oxygen vacancy (V_O_) states are ionised and temporarily relocated near the CB^11,35,55^. The extracted sub-bandgap DOS profile reflects such singly ionised oxygen vacancy (V_O_^+^) states in the deep-acceptor states and doubly ionised oxygen vacancy (V_O_^2+^) states in tail-acceptor states. If such ionised oxygen vacancies solely contribute to the photoresponse process, one could expect higher photocurrent in the 33ZTO TFT than the 50ZTO TFT irrespective of the excitation photon energy, which is not seen here. This result indicates that in addition to the V_O_^2+^ states, there are some other activated defects present in the tail states in the 50ZTO, which are also optically irreversible. It is widely considered that the oxygen vacancy defects are the major intrinsic origin of PPC effect in the oxide system. However, the effect of zinc interstitials or peroxide defects on the PPC is also proposed^56,57^. In the present case, in addition to the effect of oxygen vacancy on the PPC effect possibility of contribution from above mentioned two defects are verified. It is known that peroxide defects form under light illumination, only when a hydrogen-zinc vacancy defect complex (2H–V_Zn_) present in the system. In the present case absence of H or OH attached to the metal or oxygen ions in the XPS data (spectra for O1s and Zn2p core levels) ruled out the possibility of involvement of peroxided defect in the observed long-lasting PPC. On the other hand, due to the high tin concentration, the probability of formation of additional zinc interstitial defects due to the substitutional doping error or lattice distortion is very high in 50ZTO. The XPS data also indicate presence of high concentration of zinc interstitials (Zn_i_) in the 50ZTO. A first-principle theoretical calculation by Janotti et al.^45^ on the ZnO system found a low formation energy of the ionized Zn_i_^2+^ defects (− 0.45 eV). As a result, neutral Zn_i_ (donor defect) is easily ionized to Zn_i_^2+^ by releasing electrons and located near the CB edge^56^. Formation of Zn_i_^2+^ shallow donor within 0.15–0.2 eV below the CB was reported earlier by Kayaci et al.^58^ while studying the role of zinc interstitials and oxygen vacancies of ZnO in photocatalysis. This provides evidence that 50ZTO contains more tail states than the 33ZTO (Fig. 7b), and hence a high value of photosensitivity and long-lasting PPC. Under illumination with photon energy close to the CB, the interstitial defects release electrons and contribute to the generated photocurrent along with V_O_ defects. Just after switching off the light, the system remains in a positive bias condition at the same applied V_GS_. Because, prolonged light illumination on the oxide TFTs shifts their characteristic V_th_ towards the negative bias direction, known as light stress effect. In the present case, this shift is more than − 16 V after 2 min of light illumination. The Zn_i_^2+^ subsequently return to their previous charge state by capturing free electrons. Like the V_O_ defect, ionisation-deionisation process of the Zn_i_ is also an irreversible process due to negative formation energy of Zn_i_^2+^ (− 0.45 eV)^45,58^ which delays the recovery. Due to the high formation energy of the neutral Zn_i_ defects (6.95 eV)^45^, the deionisation process (Zn_i_^2+^ + e^−^/2e^− ^→ Zn_i_^+^/Zn_i_) during photocurrent decay is exceptionally slow. It is more likely, the Zn_i_^2+^ state captures a single electron and returns to Zn_i_^+^ state instead of directly returning to neutral Zn_i_ state. In 33ZTO, in addition to the V_O_ defect, the presence of Zn_i_ is negligible so the reset times for the UV and blue are comparable. This explains why an exceptionally slow decay is observed from the 50ZTO TFT under illumination with UV light. The photoresponse data (Fig. 4) and XPS data (Fig. 5) together further suggest that Zn_i_ defects dominants over V_O_ on the reset time when illuminated with light energy close to CB of the ZTO (tail states region). Therefore, combining the effects of V_O_ and Zn_i_ states provides a consistent explanation for the observed strong and long-lasting PPC in the 50ZTO TFT, where V_O_ and Zn_i_ states both are responsible for the long-lasting PPC.

## Conclusions

We have demonstrated that sub-bandgap light excitation near the CB of the ZTO thinfilm phototransistors leads to strong PPC with long-lasting recovery time (up to a month) with an associated transition from semiconductor ZTO to conductor-like ZTO. Furthermore, tin concentration in the ZTO ternary oxide modulates the photosensitivity as well as PPC recovery time. Under sub-bandgap excitation with UV light, photosensitivity reaches as high as ~ 10^7^ and remains close to this value for a long time after switching off the light. Photon energy dependent photoresponse studies reveal that photoexcitation energies closer to the CB result in much higher recovery time. The sub-bandgap DOS has been experimentally extracted using the DCA technique. This result provides direct evidence of the involvement of sub-bandgap tail states for strong PPC in ZTO, while deep states contribute to mild PPC. The origin of the PPC in this material is attributed to ionised oxygen vacancy and ionised zinc interstitials, which require higher excitation energy. We conclude that, oxygen vacancy and zinc interstitials both are responsible for the observed long-lasting PPC effect. This conclusion is supported by the XPS analysis result and DCA result. Such a high sensitivity with long-lasting photocurrent retention time in ZTO may find application in optical memory devices for holographic storage and non-volatile memory devices.

## Methods

### Device fabrication

We used a remote plasma reactive sputtering technique to prepare the ZTO thinfilms with 33 at% and 50 at% of tin with respect to zinc from zinc/tin alloy targets with different tin concentrations. Details of the preparation of the ZTO thinfilms and characterization results can be found elsewhere^23^. Bottom-gate, inverted staggered TFTs were fabricated by depositing an active ZTO channel layer of thickness ~ 50 nm on SiO_2_ (film) on Si (100) substrate followed by air annealing at 500 °C. The SiO_2_ layer was used as a gate dielectric and the heavily doped Si (100) substrate as the gate electrode. The top-contact was a thermally evaporated Al layer which served as the source/drain electrode. The active ZTO layer and the source/drain was patterned using a standard process for the fabrication of the TFTs with W = 1900 μm and L = 50 μm (W/L = 38). In the final stage, the device chip was mounted on a stripboard and electrical connection to the external pin connectors was made by using gold wires (~ 25 μm thick) and a wire-bonder. Fabricated TFTs are marked as 33ZTO and 50ZTO for their active channel tin concentration of 33 at% and 50 at%, respectively.

### Characterizations and electrical measurements

Elemental compositions and charging states of ions in the ZTO thinfilms were estimated from X-ray photoelectron spectroscopy (XPS) measurement (Multilab-2000, Thermo Scientific) using an Al *K*_*α*_ X-ray beam at 1486.7 eV in an ultra-high vacuum chamber. A standard UV–Vis spectrometer was employed to estimate bandgap energy of the ZTO thinfilms from the transmittance data.

All the electrical measurements of the TFTs were performed inside a light blocked dark box at room temperature using a computer controlled Keithley meter (6517B) and power supply (2230) through triax cables. The gate-to-source voltage was swept from − 25 to + 25 V at a sweep rate of 0.33 V/s. The photoexcited electrical measurement was performed by illuminating the active area of the device with different narrow-band, high-power LEDs: UV (at 365 nm), blue (at 465 nm), green (at 526 nm) and red (at 662 nm) in ON and OFF conditions. Applied V_DS_ and V_GS_ voltages were 1 V and − 7 V for the 33ZTO and 1 V and − 11 V for the 50ZTO, respectively. For DCA measurement, a voltage pulse with frequency up to 100 kHz from a pulse generator (Tektronix AFG 2021) was applied to the gate and discharge current was measured at drain using Keithley meter while source connected to the ground. The pulse width and rising/falling time were fixed to 50% of the duty cycle at a given frequency and 100 ns, respectively. Magnitudes of the flat-band voltage (V_FB_) and capacitance of the dielectric oxide layer (*C*_*g*_) were extracted precisely from the transfer-characteristic data (Fig. 2) and capacitance–voltage data (not shown) of the TFTs. The capacitance–voltage data of TFTs was measured in the standard way, by superimposing a small low-frequency ac signal (frequency 20 kHz) onto the dc bias voltage, with source and drain connected. The value of the flat-band voltage (V_FB_) was extracted from the capacitance–voltage curve following the method developed by Winter et al.^59^. This method is based on the point of inflection in the capacitance–voltage curve, which was identified from the point of intersection between the capacitance–voltage curve and its second derivative as a function of gate voltage, V_G_. The corresponding gate voltage at the point of intersection is the V_FB_ of the TFT. The extracted value of V_FB_ was cross-checked with the transfer-characteristic data and it was observed that V_FB_ appeared to be near a point from at which the drain-source current I_DS_ started to increase, as would be expected.

## Data Availability

Information on the data underpinning the results presented here, including how to access them, can be found in the Cardiff University data catalogue at 10.17035/d.2021.0139502698.
